# Novel citrus hybrids incorporating Australian lime genetics: development of HLB-tolerant citrus rootstocks and physiological changes in ‘Valencia’ sweet orange scions

**DOI:** 10.3389/fpls.2025.1614845

**Published:** 2025-06-13

**Authors:** Lamiaa M. Mahmoud, Manjul Dutt

**Affiliations:** ^1^ Citrus Research and Education Center, University of Florida, Lake Alfred, FL, United States; ^2^ Plant Breeding Graduate Program, University of Florida, Gainesville, FL, United States

**Keywords:** Huanglongbing (HLB), *Candidatus* Liberibacter asiaticus, finger lime hybrids, conventional breeding, pummelo, rootstocks

## Abstract

Citrus greening disease, or Huanglongbing (HLB), is the most destructive disease affecting citrus crops worldwide. All commercially cultivated citrus varieties are highly susceptible to HLB, and currently, no effective treatments exist. Several Australian lime species have demonstrated significant HLB tolerance, making them promising candidates for developing HLB-tolerant rootstocks and scions through conventional breeding and biotechnological approaches. Herein, we report the successful development of HLB-tolerant citrus hybrids via integrating Australian lime genetics using traditional breeding methods and protoplast fusion techniques. To test the HLB tolerance of these hybrids as rootstocks, they were clonally propagated through cuttings and divided into two groups: one grafted with *Ca*Las-free ‘Valencia’ sweet orange budwood and the other grafted with *Ca*Las-infected ‘Valencia’ budwood. The performance of these hybrids was compared to similar *Ca*Las-infected and free 'Valencia' budded onto Swingle rootstock. Total DNA was isolated from the ‘Valencia’ leaves at 6, 12, 18, and 24 months post-grafting to assess *Ca*Las titers using quantitative PCR. After two years, significantly higher Ct values (ranging from 29.11 to 35.00) was observed in ‘Valencia’ trees grafted onto the experimental hybrids than in those grafted onto Swingle, which presented a Ct value of 22.25 ± 1.11. Compared with other hybrids, the mandarin (UF304) × finger lime hybrid (MFL1-98) and pummelo × finger lime hybrid (PFL2-61) exhibited improved graft-take, enhanced growth, and lower *Ca*Las titers. Additionally, we analyzed the biochemical and molecular changes in the leaves of ‘Valencia’ grafted onto these Australian lime-derived hybrids. Biochemical analyses revealed significant alterations in chlorophyll content, starch accumulation, and levels of phenolic and flavonoid compounds. These results demonstrate the practical benefits of using Australian lime-derived hybrids as rootstocks to increase HLB tolerance in citrus, offering a promising approach for the sustainable management of HLB in commercial citrus production.

## Graphical Abstract

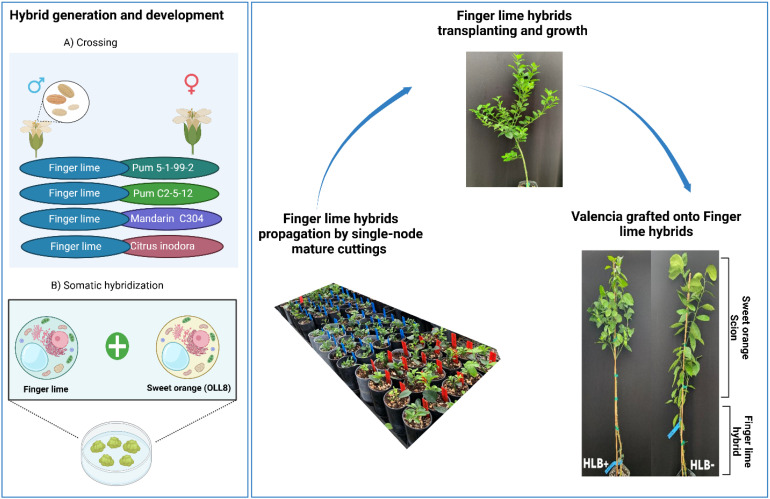

## 1 Introduction

Citrus greening disease, also known as Huanglongbing (HLB), is caused by a phloem-restricted bacteria and poses the greatest threat to global citrus production (Bové, 2006; Urbaneja et al., 2020; Pérez-Hedo et al., 2024). The most common species associated with this disease are *Candidatus* Liberibacter asiaticus (*Ca*Las), *Candidatus* Liberibacter africanus (*Ca*Laf), and *Candidatus* Liberibacter americanus (*Ca*Lam). These bacteria are transmitted by two main insect vector species: the Asian citrus psyllid (*Diaphorina citri* Kuwayama, Hemiptera: Psyllidae) and the African citrus psyllid (*Trioza erytreae* Del Guercio, Hemiptera: Triozidae).

HLB impacts all commercially grown citrus varieties, leading to drastic yield reductions, tree decline, and eventual death (Bové, 2006; Thakuria et al., 2023). Symptoms of the disease include asymmetrical leaf mosaics crossing the veins, leaf chlorosis, stunted growth, premature fruit drop, and the production of misshapen, bitter-tasting fruit (Bové, 2006; Wang and Trivedi, 2013). The rapid spread of HLB, combined with the lack of effective treatments, poses a significant challenge to the sustainability of the citrus industry worldwide. Given the complex nature of the disease, a multifaceted treatment approach is necessary. Current efforts include vector control, nutritional management, thermotherapy, and the application of antimicrobials and antibiotics to combat *Ca*Las infection. Despite these efforts, tree health continues to deteriorate, and fruit yields remain in decline, emphasizing the urgent need for more effective and sustainable solutions (Pérez-Hedo et al., 2024, 2025).

The integration of tolerant and resistant citrus relatives into breeding programs, rootstock development, and interstock applications presents a promising strategy for enhancing resilience to HLB. Some citrus species have shown resistance or tolerance to HLB. Finger lime (*Citrus australasica*), *Poncirus trifoliata*, and *C. latipes* have exhibited significant tolerance to HLB, whereas species such as desert lime (*C. glauca*) have demonstrated resistance (Ramadugu et al., 2016; Curtolo et al., 2020; Killiny et al., 2020; Weber et al., 2022; Mahmoud et al., 2024a). Recently, we investigated the response of two pummelo x finger lime hybrid siblings to HLB infection. Our findings revealed the tolerance of PFL 2-61, which presented relatively low *Ca*Las titers (Mahmoud et al., 2025).

Swingle and Reece (1967) proposed that Microcitrus and Eremocitrus (now classified as Citrus species) evolved from a common ancestor, likely resembling *C. warburgiana*, which, along with *C. papuana*, is native to New Guinea. Different evolutionary paths emerged from this ancestral form: one leading to the Australian round lime (*C. australis*), another to *C. inodora*, and a third to the Australian finger lime (*C. australasica*). In contrast, *C. glauca* rapidly evolved with distinct xerophytic adaptations suited to the Australian deserts. The chloroplast phylogeny supports the common origin of these species, confirming their monophyly, with two primary clades observed, as outlined by Bayer et al. (2009). The consistent resistance or tolerance to *Ca*Las, characterized by the absence of detectable bacterial replication in germplasms sexually compatible with Citrus, holds significant promise for utilization in sexual breeding programs. The use of hybrids of these species as rootstocks can confer improved tolerance to susceptible scions via modulation of systemic responses, increased nutrient uptake efficiency, and reduced pathogen proliferation (Castle et al., 2015). Additionally, interstocks derived from resistant germplasms could serve as a physiological barrier, mitigating the pathogen’s spread while maintaining compatibility with commercial citrus varieties.

Conventional breeding approaches, including hybridization between tolerant species and elite cultivars, can introduce durable resistance while preserving desirable fruit traits. Recent advances in genomic-assisted breeding, marker-assisted selection, and biotechnology have further enabled the precise incorporation of resistance genes into commercial citrus varieties, accelerating the development of HLB-tolerant cultivars. By leveraging these genetic resources, breeders can increase long-term sustainability and productivity in citrus orchards affected by HLB. Certain Citrus relatives have demonstrated cross-compatibility and graft compatibility with Citrus (Dutt et al., 2021; Dutt, 2022b; Mahmoud et al., 2025). The resistance or tolerance of citrus relatives to *Ca*Las can vary depending on factors such as genetic background, inoculation method, environmental conditions, plant age, number of replicates, testing environment (field vs. greenhouse), and type of plant material used (seedlings vs. mature plants, grafted vs. rooted stocks) (Alves et al., 2021). Together, these factors affect the development of tolerance in plants.

Plants have developed diverse mechanisms to tolerate stress, including physiological, biochemical, and molecular adaptations to cope with pathogen load (Kumar and Verma, 2018). These mechanisms help plants alleviate stress and sustain their growth and development in stressful environments. The generation of reactive oxygen species (ROS) within various subcellular compartments and the resulting oxidative bursts play crucial roles in enhancing tolerance to biotic stress (Balint‐Kurti, 2019). As a defensive response, a set of compounds, such as phenolic compounds, flavonoids, tannins, lignans, lignin, and monolignols, are formed through the phenylpropanoid pathway (Cheynier et al., 2013; Saltveit, 2017).

Our citrus breeding program aims to produce hybrids using HLB-tolerant citrus relatives to yield new rootstocks, interstocks, or scion varieties tolerant to HLB. In the present study, we selected six hybrids and investigated their *Ca*Las tolerance and performance as rootstocks. We assessed their graft compatibility with ‘Valencia’ sweet orange and observed their response to *Ca*Las inoculation, thereby investigating their response to infection as rootstocks. We conducted challenge inoculation by grafting *Ca*Las-infected budwood collected from the field onto these hybrids. *Ca*Las multiplication was evaluated regularly using quantitative PCR (qPCR) over 24 months after inoculation (MAI). We analyzed the susceptibility/resistance responses of the evaluated germplasms to *Ca*Las based on parameters such as the leaf chlorophyll content, foliar starch content, foliar total phenolic compound content, and flavonoid content. Additionally, we examined the performance of the trees by studying changes in the expression of defense-related transcripts identified in our prior study (Weber et al., 2022).

## 2 Materials and methods

### 2.1 Plant genotype and experiment establishment

Australian lime-derived hybrids were developed using conventional breeding and protoplast fusion techniques. For conventional breeding, the finger lime (*C. australasica*, PI 312872 accession from the USDA National Plant Germplasm System [NPGS]) was used as the male parent and crossed with red pulp pummelos (*C. maxima* (Burm.) Merr.) such as the Pum 5-1-99–2 and the Pum C2-5-12, mandarin UF304 (*C. reticulata* hybrid), or a *C. inodora* OP seedling (**Table 1**). The hybrids generated through conventional breeding were visually selected based on the color and size of the leaves (Mahmoud et al., 2025). Allotetraploid finger lime-derived hybrid was developed through somatic cell fusion, where diploid ‘OLL8’ sweet orange callus-derived protoplasts were fused with finger lime (FL) mesophyll-derived protoplasts (Dutt et al., 2021). As previously reported, the tetraploid somatic hybrid was evaluated using flow cytometry and simple sequence repeat markers (Dutt et al., 2021). The selected hybrids were propagated in a greenhouse before being transplanted into a sandy-soil field in Lake Alfred, Florida, USA, where they were naturally exposed to *Ca*Las for more than ten years through natural psyllid feeding. The trees were irrigated daily, and Florikan 12-4-8 360-day controlled-release fertilizer (FLORIKAN E.S.A. LLC, Sarasota, Florida) was applied annually in March. The hybrids used in this study were chosen based on their demonstrated tolerance for HLB and vigorous growth characteristics.

**Table 1:** List of Australian lime derived hybrids presenting the hybrids selected for investigation in the study and the parents.

Hybrid name	Ploidy	Female parent	Male parent	Origin/source
PFL1-11	2X	Pummelo 5-1-99-2	*Citrus australasica*	Sexual cross
PFL2-61	2X	Pummelo 5-1-99-2	*Citrus australasica*	Sexual cross
PFL1-3	2X	Pummelo C2-5-12	*Citrus australasica*	Sexual cross
MFL1-98	2X	Mandarin UF304	*Citrus australasica*	Sexual cross
UF SunLime	2X	*Citrus inodora* OP sdl.	*Citrus australasica*	Sexual cross
O+M2-75	4X	Sweet Orange OLL8	*Citrus australasica*	Somatic hybridization

Six selected Australian lime-derived hybrids were propagated in the mist bed via single-node mature cuttings on a Pro - Mix BX general purpose potting mix and maintained under controlled conditions for one year. Following the propagation period, the plants were transplanted into 10 × 10 × 35 cm standard square pots (“citripots”) filled with the same Pro-Mix soilless medium (Premier Tech Horticulture, Quakertown, PA, USA). They were then placed in a 50% shaded greenhouse, exposed to natural daylight and maintained under a relative humidity of 70 ± 10% and a temperature of 32 ± 2°C. The clonally propagated trees were divided into two sets. The first set was top grafted with *Ca*Las-free ‘Valencia’ sweet orange (*Citrus* × *sinensis* (L.) Osbeck) budwood. In contrast, the second set was grafted with *Ca*Las-positive ‘Valencia’ budwood obtained from HLB-infected trees (Ct values of *Ca*Las = 25 ± 3.5). The performance of those trees was compared to that of ‘Valencia’ grafted onto Swingle citrumelo, a common commercial rootstock.

### 2.2 Growth measurement

The growth index was determined via measurements of scion length, scion diameter, and rootstock diameter using a Vernier caliper and metric tape measure. Scion length was measured from the tip of the main stem to the graft union, encompassing the length of both the spring and summer shoots. The scion and rootstock diameters were measured 5 cm above and below the graft union. The experimental design comprised twelve replicates for each graft combination. Graft-take, indicating graft compatibility, was also evaluated. Initial compatibility was determined by the presence of healed grafting unions and the observation of new viable shoots. The number of live grafts was recorded for each treatment, along with the time elapsed after grafting, and the graft-take percentage relative to the total number of grafted plants was calculated. Furthermore, three plants from each combination were longitudinally sectioned with a scalpel blade at the median region of the grafted region within a 5 cm long stem segment, splitting them into two halves for sampling. The samples were stained with a 5% ferric chloride solution for 2 hours to detect phenolic compounds. The ferric chloride solution binds to the phenolic compounds in the grafting area, resulting in a visible color change. The color was then observed under a microscope (Carl Zeiss Microscopy GmbH, Göttingen, Germany) equipped with a Zeiss AxioCam ICc1. ImageJ software was used to measure the optical intensity of staining (Ruzin, 1999; Marques et al., 2018).

### 2.3 *Ca*Las assessment in ‘Valencia’ leaves and rootstock Australian lime derived hybrid roots

To assess the *Ca*Las titer in the infected leaves from the scion and the roots of the rootstock of greenhouse-grown trees, total DNA was periodically isolated from the leaf petioles and midveins of fully expanded leaves or root tissues. DNA extraction was performed using the GeneJET Plant Genomic DNA Purification Kit (Thermo Fisher Scientific, Massachusetts, USA) following the manufacturer’s instructions. The concentration of the extracted DNA was normalized to 25 ng/μL before conducting quantitative PCR (qPCR). Quantitative PCR was carried out using a QuantStudio™ 3 System (Thermo Fisher Scientific). The *Ca*Las genomic DNA was tested using TaqMan™ Gene Expression Master Mix and the CQUL primers (**Supplementary Table 1**), designed to amplify a segment of the *Ca*Las rplJ/rplL ribosomal protein-encoding gene (Wang et al., 2006). *Ca*Las multiplication was evaluated regularly over 24 MAI at 6, 12, 18, and 24 MAI with *Ca*Las-infected or *Ca*Las-free (control) trees.

### 2.4 Physiological and biochemical variables

Fifteen mature leaves from ‘Valencia’ plants were harvested, frozen, and ground in liquid nitrogen. Each hybrid was sampled in six biological replicates, and the ground leaf material was stored at -20°C until biochemical assays were performed. Chlorophyll *a*, chlorophyll *b*, and total chlorophyll levels were determined by measuring the absorbance at different wavelengths (665 nm for chlorophyll *a* (Chl *a*) and 653 nm for chlorophyll *b* (Chl *b*)). Chlorophyll *a*, chlorophyll *b*, and total chlorophyll contents were estimated (Lichtenthaler and Wellburn, 1983).

The starch content was determined as described previously by Rosales and Burns (2011) with slight modifications. Fresh tissues (100 mg) were ground to a powder, suspended, and homogenized in 700 μl of distilled water. Leaf samples and a standard solution were boiled in water for 10 min and then transferred to cold water to cool. The samples were vortexed and then centrifuged for 2 min at 6000 rpm. Aliquots of the supernatant were collected in new tubes. Three hundred microliters of the supernatant were subjected to extraction with 900 μl of 100% ethanol. The mixture was vortexed and centrifuged for 10 min at 10000 rpm. The supernatant was discarded, and 1 ml of distilled water was added to dissolve the pellet. Fifty microliters of KI: I2 (8 mM: 50 mM) was added. The starch content was quantified by monitoring the color change with a spectrophotometer at 594 nm. Rice starch (Sigma Aldrich, St. Louis, MO) was used as a standard.

The total phenolic compounds were estimated according to Singleton and Rossi (1965). In brief, 100 mg of fresh leaf tissue was subjected to extraction in 1 ml of 80% ethanol and then centrifuged for 20 min at 10000 rpm. The TPC extract was centrifuged, and 100 µL of Folin reagent (1:10) was mixed with the leaf extract. After vortexing and 5 min of incubation at room temperature, the reaction was initiated by adding 300 mL of 20% sodium carbonate (Na_2_CO_3_) to the extract. The color change was recorded after 60 min by measuring the absorbance at 650 nm. The standard curve for phenol was prepared by measuring 1 mL each of a series of gallic acid solutions in ethanol at different concentrations from 0 to 1.00 mg/mL. The phenol contents are expressed as mg of gallic acid per 100 g of tissue fresh weight.

The total flavonoid content in the leaf samples was estimated using a colorimetric assay with aluminum chloride (Aktumsek et al., 2013). The methanolic extract (50 µL) was diluted in 200 µL of distilled water, and the diluted samples were mixed with 60 µL of 5% sodium nitrite (NaNO_2_) solution and 60 µL of 10% aluminum chloride (AlCl_3_) solution. After a 5-minute incubation at room temperature, 400 µL of 1 M sodium hydroxide (NaOH) and 1.230 mL of distilled water were added before vortexing. The absorbance of the reaction mixture was measured at 510 nm. A standard curve was created using catechin standard solutions (0–200 ppm), and the content of total flavonoids is expressed as mg catechin equivalents per 100 mg of fresh weight.

### 2.5 Gene expression assessment

RNA was isolated according to the manufacturer’s protocol from 100 mg of finely ground leaf tissue via a Direct-zol™ RNA Miniprep Kit. qPCR was performed with a final volume of 10 μl using the SYBR Green Kit according to the manufacturer’s instructions. Each sample was tested twice in twelve replicates. The Ct value of the PCR curve was analyzed and compared with that of Val/SW as a control. The relative expression levels of the selected genes were calculated by the 2^−ΔΔCT^ method (Livak and Schmittgen, 2001) and the calculation of Log2 fold change compared to ‘Valencia’ grafted onto Swingle rootstock using RStudio. The actin housekeeping gene was used as an endogenous control. The primer sequences of the genes evaluated in this study are outlined in **Supplementary Table 2**.

### 2.6 Statistical analysis

To investigate the performance of ‘Valencia’ sweet orange grafted on the Australian lime-derived hybrids, analysis of variance (ANOVA) was conducted in JMP Pro version 17 (SAS Institute, Cary, NC, USA) and RStudio (version 2024.12.1 Build 563, Posit Software, PBC). The *Ca*Las infection and scion/rootstock combinations were examined via a factorial experiment with two factors (*Ca*Las– or *Ca*Las + infection) and seven scion/rootstock combinations. For gene expression analysis, data collected under *Ca*Las+ conditions were evaluated using one-way ANOVA to compare relative gene expression among graft combinations, with ‘Valencia’ on Swingle rootstock as the control. ANOVA followed by the *post hoc* Tukey–Kramer honestly significant difference (HSD) test was used. Differences were considered significant when the *p* value was less than 0.05%.

## 3 Results

### 3.1 Graft compatibility on Australian lime-derived hybrids as rootstocks

**Figure 1:**
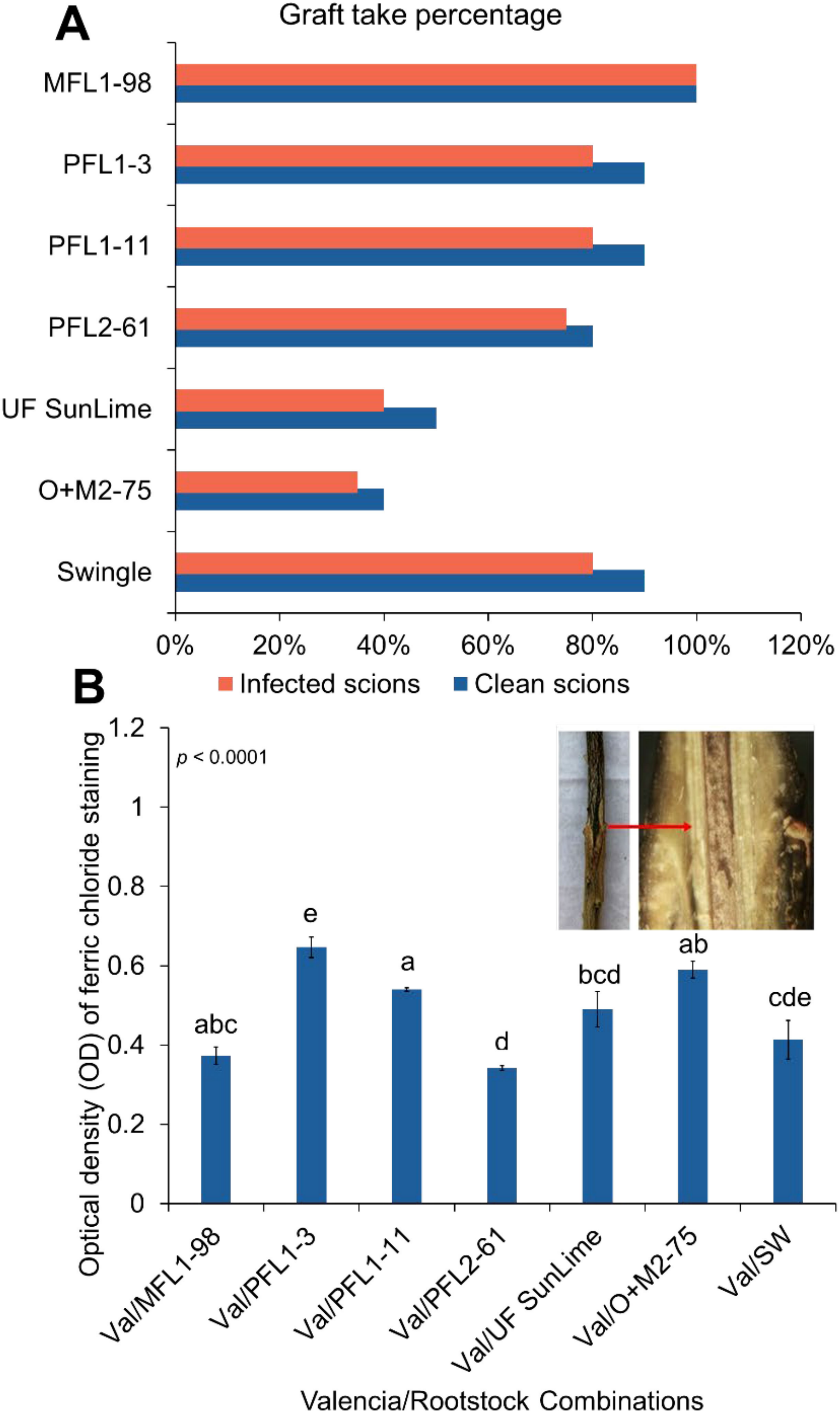
**(A)** Graft-take or grafting success rates of ‘Valencia’ sweet orange on Australian lime-derived hybrids with *Ca*Las− and *Ca*Las+ budwood. **(B)** Staining intensity measurements for phenolic compounds in various citrus scion/rootstock combinations. The image on the right side of panel **(B)** shows longitudinal sectioning of the MFL1–98 combination at the median graft region within a 5 cm stem segment.

‘Valencia’ sweet orange exhibited graft compatibility with Australian lime-derived hybrids, with grafting success rates ranging from 80% to 100% when *Ca*Las - budwood was used and 75% to 100% when *Ca*Las+ budwood was used. However, two hybrids, O+M2–75 and UF SunLime, presented lower grafting success rates of 40% and 50%, respectively. When grafted with *Ca*Las+ ‘Valencia’ budwood, the percentage of grafted plants further decreased to 35% and 40% in these hybrids, respectively (**Figure 1A**). To assess the accumulation of phenolic compounds at the graft union in infected trees, we applied a 5% ferric chloride solution to longitudinal sections of the grafting union, and the staining intensity was estimated in the infected trees. The mean optical density (OD) values varied among the hybrids (*p* < 0.0001) (**Figure 1B**). Val/PFL 2–61 and Val/MFL1–98 presented the lowest OD values (0.34 and 0.37, respectively), whereas PFL 1–3 presented the highest OD at 0.6467 (**Figure 1B**).

### 3.2 ‘Valencia’ grafted onto Australian lime derived hybrids exhibited improved growth performance

The growth performance of ‘Valencia’ orange grafted onto Australian lime-derived hybrids was assessed under both *Ca*Las-free (HLB−) and *Ca*Las-infection (HLB+) conditions at 12 and 24 months post grafting (**Table 2**). The results revealed significant variations in plant vigor among the different rootstock combinations. At 12 months post-grafting, Val/PFL2–61 and Val/MFL1–98 presented the most significant growth across all the measured parameters, with a height of 67.17 cm, a scion diameter of 5.71 cm, and a rootstock diameter of 9.70 cm under *Ca*Las-free (HLB−) conditions. Similarly, Val/MFL1–98 presented a comparable plant height of 67.13 cm, with a slightly larger scion diameter of 6.16 cm and a rootstock diameter of 9.14 cm. Under *Ca*Las-infection (HLB+) conditions, a general decline in growth was observed across all rootstock combinations; however, Val/MFL1–98 maintained the highest growth among the infected trees, with a plant height of 65.32 cm, a scion diameter of 5.90 cm, and a rootstock diameter of 9.05 cm.

**Table 2:** Characteristics of ‘Valencia’ as a scion and the Australian lime-derived hybrids as rootstocks.

Code	Rootstock	Scion height		Scion diameter		Rootstock diameter	
		HLB-	HLB+	HLB-	HLB+	HLB-	HLB+
12-months - post grafting							
Val/PFL1-11	5-1-99–2 X FL -(Hybrid 1)	43.17	25.33	5.34	5.39	8.00	8.99
Val/PFL2-61	5-1-99–2 X FL -(Hybrid 2)	67.17	53.83	5.71	5.31	9.70	9.90
Val/PFL1-3	C2-5–12 X FL	32.33	30.87	3.47	3.07	6.42	6.69
Val/MFL1-98	UF304 X FL	67.13	65.32	6.16	5.90	9.14	9.05
Val/O+M2-75	OLL8+FL	37.00	44.00	3.83	3.27	6.41	5.58
Val/UF SunLime	UF SunLime	40.00	38.67	4.20	3.43	6.07	6.12
Val/SW	Swingle	55.67	47.83	5.18	4.80	8.00	7.83
24-months - post grafting							
Val/PFL1-11	5-1-99–2 X FL -(Hybrid 1)	86.22	85.57	8.80	8.15	12.35	11.70
Val/PFL2-61	5-1-99–2 X FL -(Hybrid 2)	124.71	94.05	12.55	10.46	12.55	10.73
Val/PFL1-3	C2-5–12 X FL	71.77	64.37	6.69	5.71	9.26	7.00
Val/MFL1-98	UF304 X FL	101.76	96.66	10.20	9.84	10.98	10.68
Val/O+M2-75	OLL8+FL	59.29	57.79	6.31	5.78	7.94	7.26
Val/UF SunLime	UF SunLime	86.61	72.90	7.51	6.73	7.96	7.25
Val/SW	Swingle	86.78	73.03	9.28	7.85	12.99	11.13

Other rootstock combinations, including Val/SW (Swingle), Val/PFL1-11, and Val/UF SunLime, also presented moderate vigor at 12 months. The plant height of Val/SW reached 55.67 cm (HLB−) and 47.83 cm (HLB+), whereas that of Val/UF SunLime reached 40.00 cm (HLB−) and 38.67 cm (HLB+). In contrast, Val/PFL1–3 presented the lowest growth in the first year, with heights of 32.33 cm (HLB−) and 30.87 cm (HLB+), along with the smallest scion and rootstock diameters among all the tested hybrids. At 24 months post grafting, Val/PFL2–61 and Val/MFL1–98 continued to exhibit the most vigorous growth. Val/PFL2–61 presented the greatest plant height (124.71 cm in HLB− and 94.05 cm in HLB+), along with substantial scion (12.55 cm, 10.46 cm) and rootstock (12.55 cm, 10.73 cm) diameters. Val/MFL1–98 maintained strong growth performance, with plant heights reaching 101.76 cm (HLB−) and 96.66 cm (HLB+), with scion diameters of 10.20 cm and 9.84 cm, respectively. Conversely, Val/O+M2–75 exhibited the lowest overall growth at 24 months, with plant heights of 59.29 cm (HLB−) and 57.79 cm (HLB+), as well as the smallest rootstock diameter among all combinations (7.94 cm and 7.26 cm, respectively). Additionally, Val/PFL1-3, despite showing some improvement in growth, remained among the least vigorous combinations, with plant heights of 71.77 cm (HLB−) and 64.37 cm (HLB+).

### 3.3 ‘Valencia’ grafted onto Australian lime derived hybrid rootstocks exhibited a lower *Ca*Las titer

**Figure 2:**
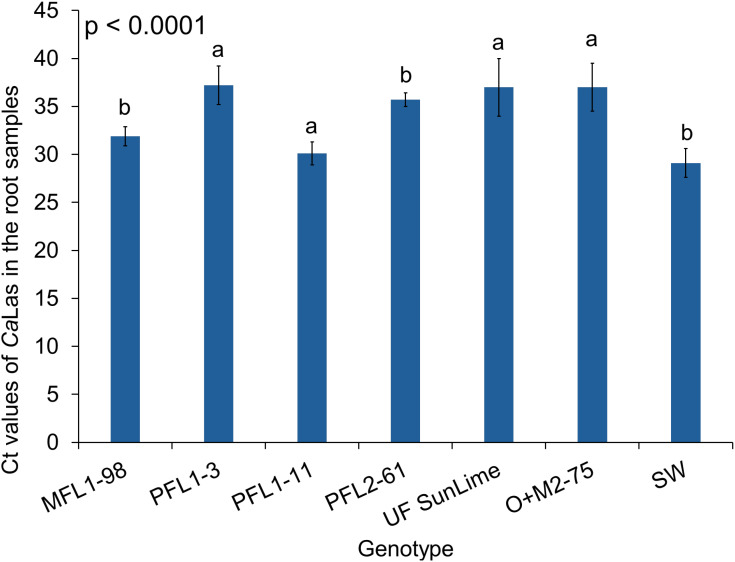
CT values of *Ca*Las bacterial titers in the roots after one year of infection. The data are presented as the means of CT values ± standard deviations. Different letters indicate significant differences across cultivars at each time point (*p* < 0.05).

*Ca*Las bacterial titers were evaluated in ‘Valencia’ grafted onto various rootstocks at four sampling periods (6, 12, 18, and 24 MAI) (**Table 3**). At 6 MAI, the Ct values ranged from 21.1 to 23.3 in ‘Valencia’ leaves, with Val/PFL2–61 showing the lowest Ct value (21.1), whereas Val/SW presented the highest Ct value (23.3). At 12 MAI, the Ct values varied significantly (*p* < 0.0001), with Val/PFL1–3 exhibiting the highest Ct value (35.3), whereas Val/PFL1–11 had the lowest Ct value (22.5). Similarly, Val/UF SunLime, Val/O+M2-75, and Val/MFL1–98 displayed higher Ct values (33.5, 31.6, and 30.8, respectively) than Val/SW (24.2). At 18 MAI, the Ct values continued to reflect differences in the bacterial titer (*p* < 0.0001). Val/PFL1–3 maintained a high Ct value (33.0), whereas Val/PFL2-61, Val/PFL1-11, and Val/SW presented the lowest Ct values (26.4, 22.3, and 24.2, respectively). At 24 MAI, the Ct values remained significantly different across rootstocks (*p* < 0.0001). Val/PFL1–3 presented the highest Ct value of 35.1, followed by Val/MFL1-98 (30.6), Val/O+M2-75 (25.0), Val/PFL1-11 (22.4), and Val/UF SunLime (22.1). Additionally, the Ct values of the root samples from the hybrids ranged from 30.1 to 37.7, with all the hybrids testing negative for *Ca*Las in the roots. However, PF1–11 and Swingle presented the lowest Ct values in the root samples (30.1 and 29.1, respectively) (**Figure 2**).

**Table 3:** Ct values of *Ca*Las bacterial titers in ‘Valencia’ leaves at different time points following qPCR analysis.

Code	6 MAI*	12 MAI	18 MAI	24 MAI
Val/PFL1-11	22.6 ± 0.3^ab^	22.5 ± 0.6^c^	22.3 ± 0.1^c^	22.4 ± 0.3^c^
Val/PFL2-61	21.1 ± 0.1^c^	24.5 ± 1.9^bc^	26.4 ± 1.2^bc^	28.5 ± 2.2^b^
Val/PFL1-3	21.8 ± 0.1^bc^	35.3 ± 0.8^a^	33.0 ± 0.9^a^	35.1 ± 0.7^a^
Val/MFL1-98	21.9 ± 0.2^abc^	30.8 ± 2.7^ab^	31.0 ± 2.2^ab^	30.6 ± 2.5^ab^
Val/O+M2-75	21.8 ± 0.2^bc^	31.6 ± 0.5^a^	25.8 ± 0.3^c^	25.0 ± 0.6^bc^
Val/UF SunLime	22.6 ± 0.4^ab^	33.5 ± 0.6^a^	26.9 ± 0.3^bc^	22.1 ± 0.1^c^
Val/SW	23.3 ± 0.5^a^	24.2 ± 1.5^c^	24.2 ± 0.8^c^	25.8 ± 0.9^bc^
*p* value	0.0004	<0.0001	<0.0001	<0.0001

*MAI, Months after infection. The data is presented as the means of CT values ± standard deviations. Different letters indicate significant differences across cultivars at each time point (*p* < 0.05).

### 3.4 Biochemical response of ‘Valencia’ sweet orange grafted onto Australian lime derived hybrids to *Ca*Las infection

**Figure 3:**
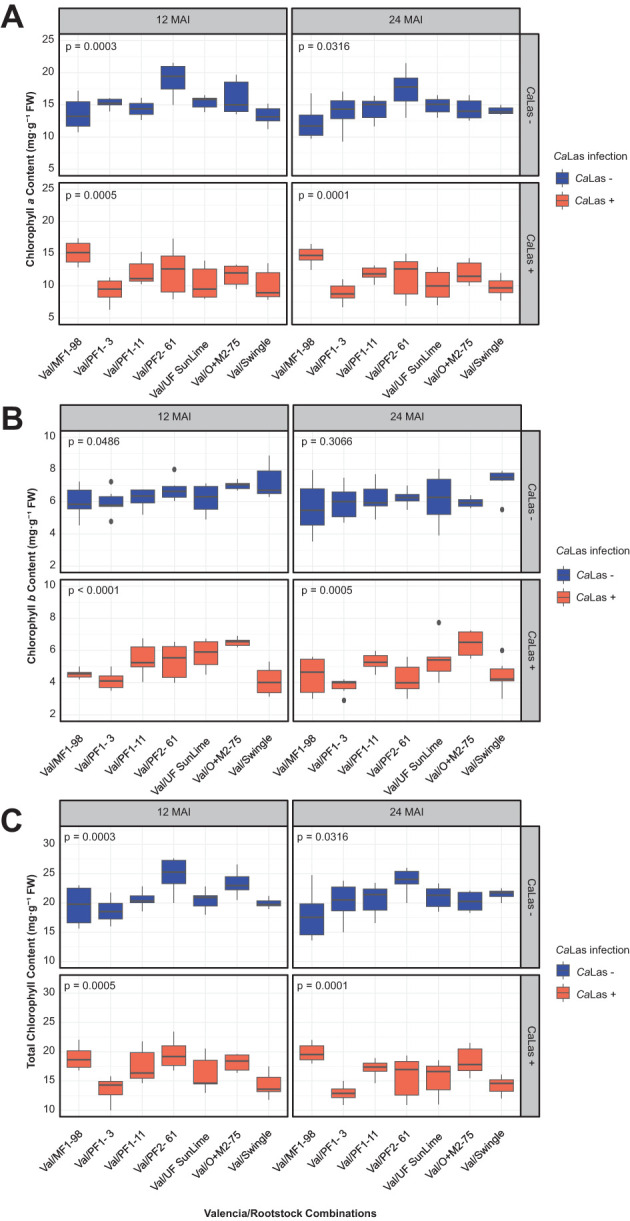
Changes in foliar chlorophyll content and starch accumulation in ‘Valencia’ sweet orange grafted onto Australian lime derived hybrids. **(A)** Chlorophyll *a* concentration (mg g^-1^ FW), **(B)** chlorophyll *b* concentration (mg g^-1^ FW), and **(C)** total chlorophyll content (mg g^-1^ FW). The data were recorded in *Ca*Las – (grafted with healthy budwoods) and *Ca*Las + (grafted with infected budwoods) at 12 and 24 months after inoculation (MAI). The box plot represents six replicates, and *p* values were determined by one-way ANOVA among the scion/rootstock combinations under each condition.

Analysis of the chlorophyll content revealed significant differences across the hybrids, with both chlorophyll *a* (Chl *a*) and chlorophyll *b* (Chl *b*) levels significantly reduced in plants infected with *Ca*Las (*p* < 0.05) (**Figure 3**; **Table 4**). At 12 MAI, the infected plants exhibited consistently lower Chl *a* values, with the most pronounced reduction observed in Val/PF1-3 (9.27 mg/g), compared to its non-infected counterpart (15.26 mg/g). Similarly, reductions were recorded in Val/UF SunLime (from 15.40 to 10.40 mg/g), Val/PF2-61 (from 18.94 to 12.28 mg/g), and Val/O+M2-75 (from 16.10 to 11.64 mg/g). Among all genotypes, Val/PF2–61 had the highest Chl *a* content under non-infected conditions, while Val/PF1–3 showed the lowest Chl *a* content under infection. At 24 MAI, the trend continued, with *Ca*Las-infected plants exhibiting further Chl *a* reduction in most genotypes. Val/PF1–3 again had an obvious drop from 13.91 mg/g(non-infected) to 8.92 mg/g (infected). Val/PF2–61 and Val/UF SunLime also showed significant declines (17.42 to 11.46 mg/g, and 14.88 to 10.07 mg/g, respectively). Val/Swingle and Val/O+M2–75 maintained relatively stable Chl *a* levels between 12 and 24 MAI but still exhibited a consistent decline in the presence of infection (**Figure 3A**).

**Table 4:** Analysis of variance (ANOVA) of the biochemical response of ‘Valencia’ sweet orange grafted onto Australian lime-derived hybrids to *Ca*Las infection at two time points.

Variables	Source	DF	Sum Sq	Mean Sq	F value	Significance
Scion diameter	Rootstock	4	146.98	36.74	32.915	***
	*Ca*Las Infection	1	2.02	2.02	1.81	ns
	Time	1	201.4	201.4	180.411	***
	Rootstock × Infection	3	0.09	0.03	0.027	ns
	Rootstock × Time	3	4.51	1.5	1.346	ns
	Infection × Time	1	1.53	1.53	1.374	ns
	Rootstock × Infection × Time	2	1.3	0.65	0.581	ns
	Residuals	65	72.56	1.12		
Rootstock diameter	Rootstock	4	149.54	37.38	23.637	***
	*Ca*Las Infection	1	0.33	0.33	0.211	ns
	Time	1	81.15	81.15	51.31	***
	Rootstock × Infection	3	1.48	0.49	0.312	ns
	Rootstock × Time	3	11.8	3.93	2.486	ns
	Infection × Time	1	2.37	2.37	1.499	ns
	Rootstock × Infection × Time	2	1.49	0.75	0.472	ns
	Residuals	65	102.8	1.58		
Plant height	Rootstock	4	18067	4517	19.764	***
	*Ca*Las Infection	1	96	96	0.421	ns
	Time	1	25797	25797	112.877	***
	Rootstock × Infection	3	1096	365	1.599	ns
	Rootstock × Time	3	1300	433	1.897	ns
	Infection × Time	1	399	399	1.745	ns
	Rootstock × Infection × Time	2	175	87	0.382	ns
	Residuals	65	14855	229		
Chlorophyll a	Rootstock	6	191	31.8	6.835	***
	*Ca*Las Infection	1	13.1	13.1	2.808	ns
	Time	1	506.3	506.3	108.71	***
	Rootstock × Infection	6	9.1	1.5	0.326	ns
	Rootstock × Time	6	269.3	44.9	9.638	***
	Infection × Time	1	2.9	2.9	0.614	ns
	Rootstock × Infection × Time	6	9.1	1.5	0.325	ns
Chlorophyll b	Rootstock	6	35.92	5.99	6.963	***
	*Ca*Las Infection	1	2.94	2.94	3.417	ns
	Time	1	76.32	76.32	88.786	***
	Rootstock × Infection	6	3.85	0.64	0.747	ns
	Rootstock × Time	6	35.14	5.86	6.814	***
	Infection × Time	1	0	0	0.001	ns
	Rootstock × Infection × Time	6	2.55	0.43	0.495	ns
Total Chlorophyll	Rootstock	6	340.5	56.7	9.755	***
	*Ca*Las Infection	1	9.4	9.4	1.609	ns
	Time	1	786.9	786.9	135.279	***
	Rootstock × Infection	6	43.5	7.2	1.246	ns
	Rootstock × Time	6	242.1	40.3	6.935	***
	Infection × Time	1	1.3	1.3	0.23	ns
	Rootstock × Infection × Time	6	50.3	8.4	1.441	ns
Starch	Rootstock	6	6.584	1.097	7.054	***
	*Ca*Las Infection	1	6.086	6.086	39.122	***
	Time	1	0.733	0.733	4.713	*
	Rootstock × Infection	6	0.056	0.009	0.06	ns
	Rootstock × Time	6	2.59	0.432	2.775	*
	Infection × Time	1	0.034	0.034	0.216	ns
	Rootstock × Infection × Time	6	0.047	0.008	0.051	ns
Total Phenolic Content	Rootstock	6	128228	21371	13.042	***
	*Ca*Las Infection	1	1274	1274	0.778	ns
	Time	1	7105	7105	4.336	*
	Rootstock × Infection	6	17554	2926	1.785	ns
	Rootstock × Time	6	61327	10221	6.238	***
	Infection × Time	1	17	17	0.011	ns
	Rootstock × Infection × Time	6	12271	2045	1.248	ns
Total Flavonoid Content	Rootstock	6	259754	43292	37.988	***
	*Ca*Las Infection	1	1021	1021	0.896	ns
	Time	1	22698	22698	19.917	***
	Rootstock × Infection	6	27047	4508	3.956	**
	Rootstock × Time	6	47584	7931	6.959	***
	Infection × Time	1	515	515	0.452	ns
	Rootstock × Infection × Time	6	38227	6371	5.591	***

This table displays the degrees of freedom (DF), sum of squares (Sum Sq), mean squares (Mean Sq), *F* values, and *p* values. Significant codes: 0 ‘***’ 0.001 ‘**’ 0.01 ‘*’ < 0.05 ‘ns’ not significant.

Across all genotypes, *Ca*Las infection consistently reduced Chl *b* content compared to uninfected controls. At 12 MAI, infected plants exhibited lower Chl *b* levels. For instance, Val/Swingle decreased from 7.22 mg/g (uninfected) to 4.11 mg/g (infected), Val/PF2–61 declined from 6.75 to 5.33 mg/g, and Val/MF1–98 dropped from 5.98 to 4.54 mg/g. The genotype with the lowest reduction was Val/O+M2-75, with infected plants retaining a relatively higher Chl *b* level (6.52 mg/g vs. 7.02 mg/g uninfected). In contrast, Val/PF1–3 and Val/UF SunLime showed a significant decline of over 1.8 mg/g. At 24 MAI, the downward trend in Chl *b* content continued in infected plants. Val/PF1–3 declined from 5.96 mg/g (uninfected) to 3.78 mg/g (infected), Val/PF2–61 from 6.25 to 4.23 mg/g, and Val/MF1–98 from 5.65 to 4.43 mg/g (**Figure 3B**).

Total chlorophyll (T Chl) content was significantly reduced in *Ca*Las-infected plants compared to uninfected controls across all genotypes and both time points (12 and 24 MAI). The extent of chlorophyll loss varies depending on the genotype. At 12 MAI, the greatest reductions were observed in Val/PF2-61 (19.56 mg/g in infected vs. 24.79 mg/g in uninfected), Val/O+M2-75 (18.16 mg/g vs. 23.33 mg/g), and Val/PF1-3 (13.60 mg/g vs. 18.67 mg/g). In contrast, Val/MF1–98 showed minimal reduction, with 18.95 mg/g in infected plants compared to 19.53 mg/g in uninfected ones. By 24 MAI, chlorophyll loss became more pronounced in most genotypes. The most affected combinations included Val/PF2-61 (15.69 mg/g infected vs. 23.81 mg/g uninfected), Val/PF1-3 (12.92 mg/g vs. 20.22 mg/g), and Val/Swingle (14.25 mg/g vs. 21.50 mg/g). Val/MF1–98 maintained relatively higher chlorophyll levels under infection, with a slight difference between infected (19.81 mg/g) and uninfected (17.94 mg/g) plants (**Figure 3C**).

**Figure 4:**
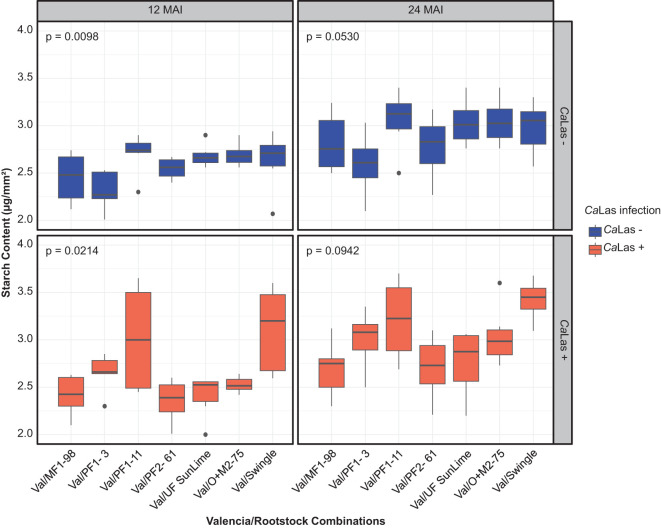
Changes in starch accumulation in the leaves (µg mm^-2^). The data were recorded in *Ca*Las – (grafted with healthy budwoods) and *Ca*Las + (grafted with infected budwoods) at 12 and 24 months after inoculation (MAI). The box plot represents six replicates, and *p* values were determined by one-way ANOVA among the scion/rootstock combinations under each condition.

Starch content was elevated in *Ca*Las-infected plants compared to uninfected controls at both 12 and 24 months after inoculation (MAI), with clear variation among genotypes. At 12 MAI, the highest starch accumulation among infected plants was observed in Val/PF1-11 (3.18 µg/mm^2^), followed by Val/Swingle (2.82 µg/mm^2^) and Val/PF1-3 (2.66 µg/mm^2^). In contrast, the lowest values were recorded in Val/PF2-61 (2.36 µg/mm^2^), Val/MF1-98 (2.42 µg/mm^2^), and Val/UF SunLime (2.41 µg/mm^2^). By 24 MAI, starch levels significantly increased in most infected combinations, with the highest content measured in Val/PF1-11 (3.53 µg/mg), Val/Swingle (3.28 µg/mg), and Val/O+M2-75 (3.04 µg/mg). Meanwhile, uninfected control plants maintained relatively stable starch levels across both time points (**Figure 4** and **Table 4**).

### 3.5 Total phenolic content and total flavonoid content of ‘Valencia’ sweet orange grafted onto Australian lime derived hybrids

The ANOVA for total phenolic content (TPC) and total flavonoid content (TFC) revealed distinct effects of rootstock, time, and their interactions (**Table 4**). For TPC, significant main effects were observed for rootstock (F = 13.042, *p* < 0.001) and time (F = 4.336, *p* < 0.05). A significant rootstock × time interaction was also detected (F = 6.238, *p* < 0.001). In contrast, *Ca*Las infection alone did not significantly affect TPC levels (F = 0.778, *p* = 0.378), and none of its interactions with rootstock or time reached statistical significance.

For TFC, highly significant effects were also observed for rootstock (F = 37.988, *p* < 0.001) and time (F = 19.917, *p* < 0.001). Additionally, interaction terms were significant, including rootstock × infection (F = 3.956, *p* < 0.01), rootstock × time (F = 6.959, *p* < 0.001), and rootstock × infection × time (F = 5.591, *p* < 0.001). Similar to TPC, infection alone did not result in a significant main effect on TFC (F = 0.896, *p* = 0.344), indicating the importance of genotype-specific responses over infection status alone.

**Figure 5:**
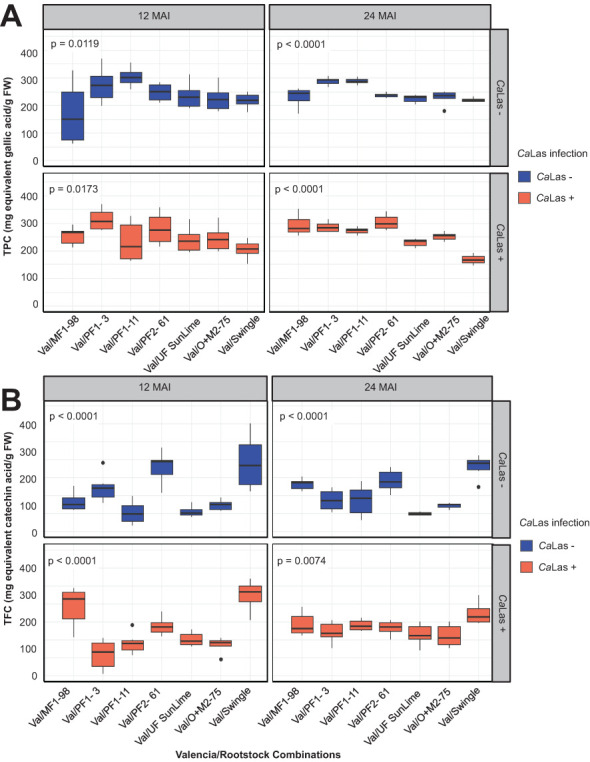
Changes in total phenolic content **(A)** and total flavonoid content **(B)** in ‘Valencia’ sweet orange grafted onto Australian lime-derived hybrids. The data were recorded in *Ca*Las – (grafted with healthy budwoods) and *Ca*Las + (grafted with infected budwoods) at 12 and 24 months after inoculation (MAI). The box plot represents six replicates, and *p* values were determined by one-way ANOVA among the scion/rootstock combinations under each condition.

Despite the absence of significant main effects of infection, both TPC and TFC levels were higher in *Ca*Las-infected plants compared to non-infected controls at both 12 and 24 months after inoculation (MAI) (**Table 4**). At 12 MAI, TPC in infected plants ranged from 205.0 to 312.5 µg/g, with the lowest level observed in Val/Swingle and the highest in Val/PF1-3. In non-infected plants, TPC values ranged from 169.7 to 303.3 µg/g. By 24 MAI, TPC levels in infected plants ranged between 167.6 and 303.1 µg/g, with Val/Swingle exhibiting the lowest concentration and Val/PF2–61 the highest. In non-infected counterparts, the TPC range was 220.0 to 287.9 µg/g, with the lowest detected in Val/PF2–61 and the highest in Val/PF1-3 (**Figure 5A**). These results indicate that while infection may induce phenolic accumulation in some genotypes, the magnitude and direction of change are strongly dependent on the specific rootstock.

For TFC, non-infected plants showed a TFC range of 131.9 to 291.9 µg/g, with Val/PF1–11 and Val/Swingle showing the lowest and highest levels, respectively. However, the infected plants at 12 MAI displayed values ranging from 144.4 to 323.6 µg/g. The lowest level was recorded in Val/PF1-3, while the highest was in Val/Swingle. At 24 MAI, TFC content of the non-infected plants ranged between 149.5 to 279.96 µg/g, with Val/UF SunLime having the lowest value and Val/Swingle the highest (**Figure 5B**). Whereas the values in the infected plants ranged from 210.2 to 272.8 µg/g.

### 3.6 ‘Valencia’ grafted onto Australian lime-derived hybrids exhibited differential expression of defense-related genes

**Figure 6:**
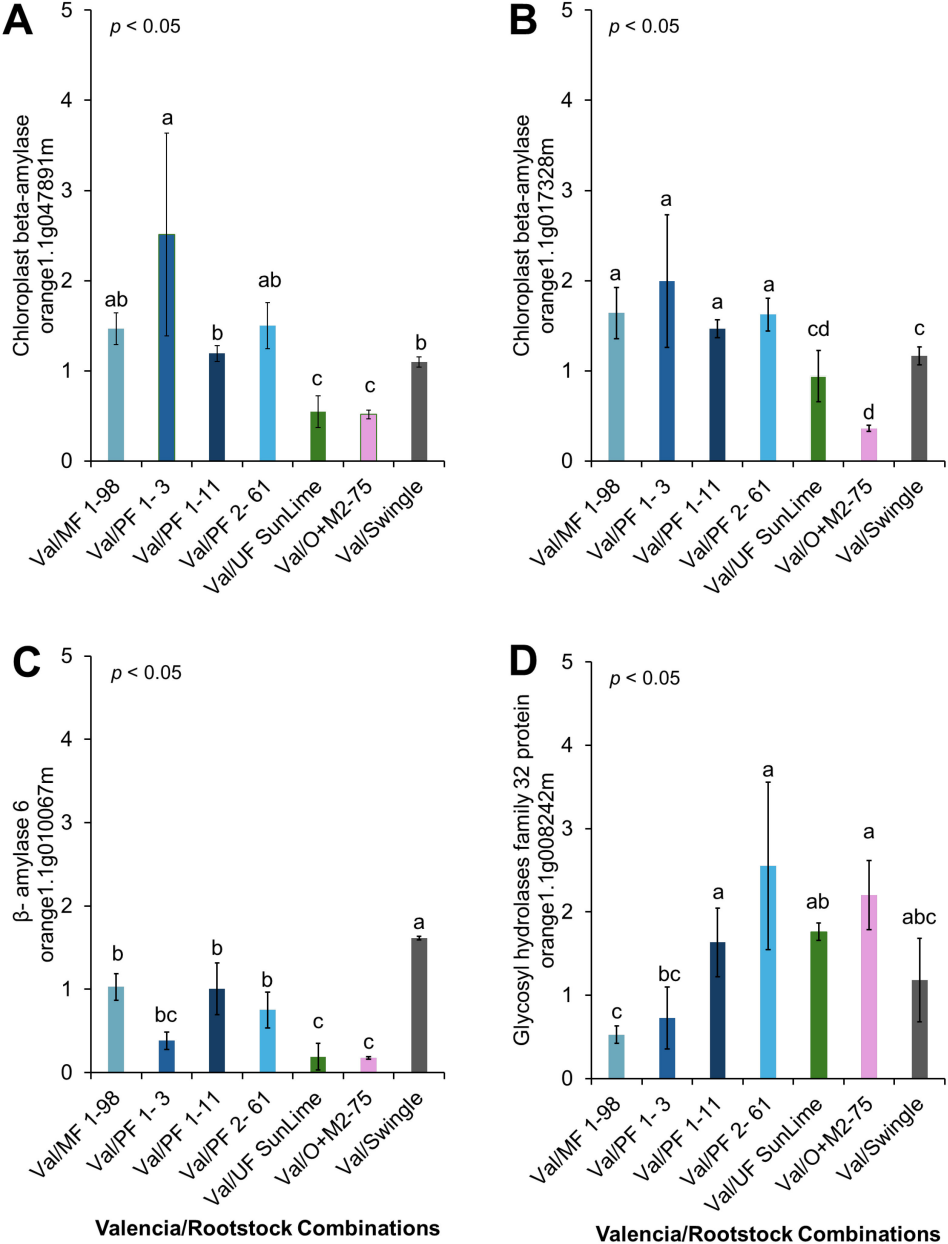
Relative expression of selected genes involved in carbohydrate metabolism. **(A)** *Chloroplast beta-amylase* (orange1.1g047891m), **(B)** *Chloroplast beta-amylase* (orange1.1g017328m), **(C)** *β-amylase 6* (orange1.1g010067m), **(D)** *Glycosyl hydrolase family 32* protein (orange1.1g008242m). Bars represent the means ± standard errors. Different letters indicate significant differences across cultivars (*p* < 0.05).

**Figure 7:**
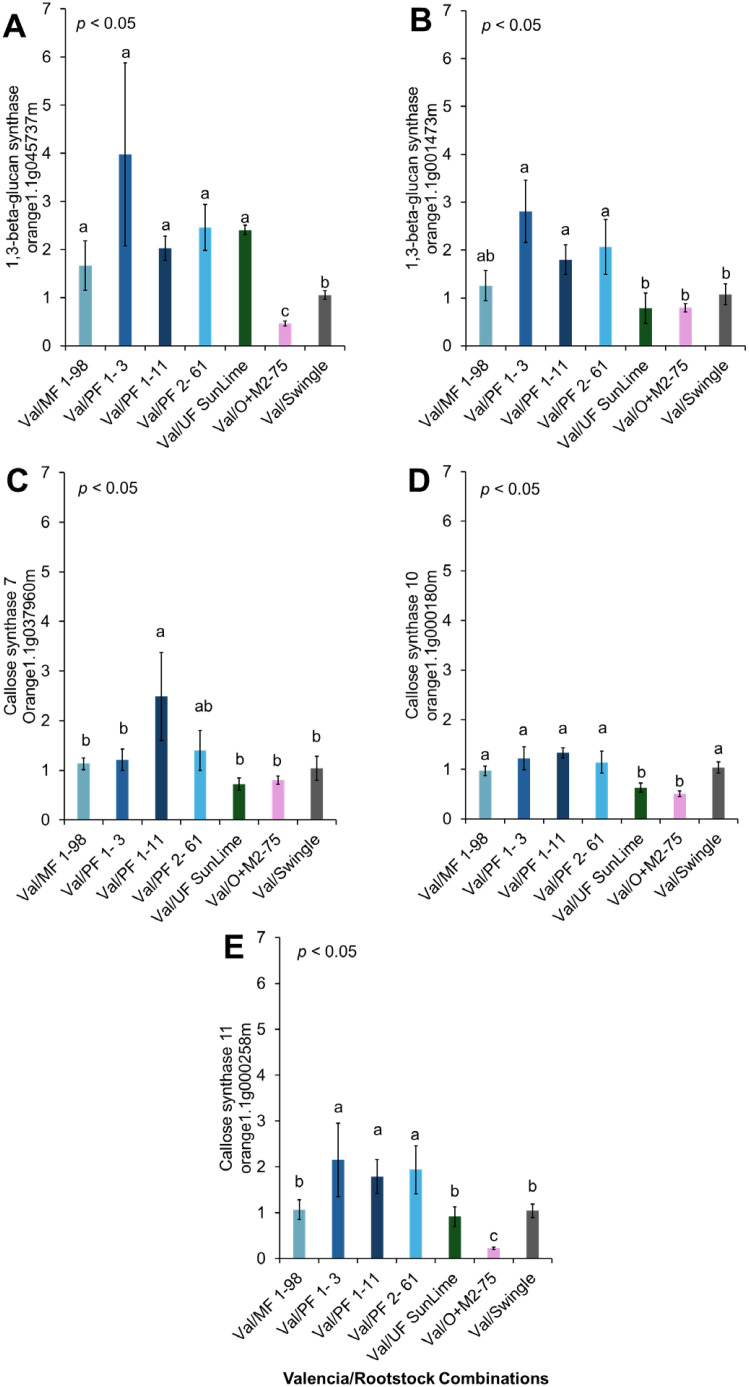
Relative expression of selected genes associated with callose synthases. **(A)** *1,3-beta-glucan synthase* (orange1.1g045737m), **(B)** *1,3-beta-glucan synthase* (orange1.1g001473m), **(C)** *Callose synthase 7* (Orange1.1g037960m), **(D)** *Callose synthase 10* (orange1.1g000180m), **(E)** *Callose synthase 11* (orange1.1g000258m). Bars represent the means ± standard errors. Different letters indicate significant differences across cultivars (*p* < 0.05).

**Figure 8:**
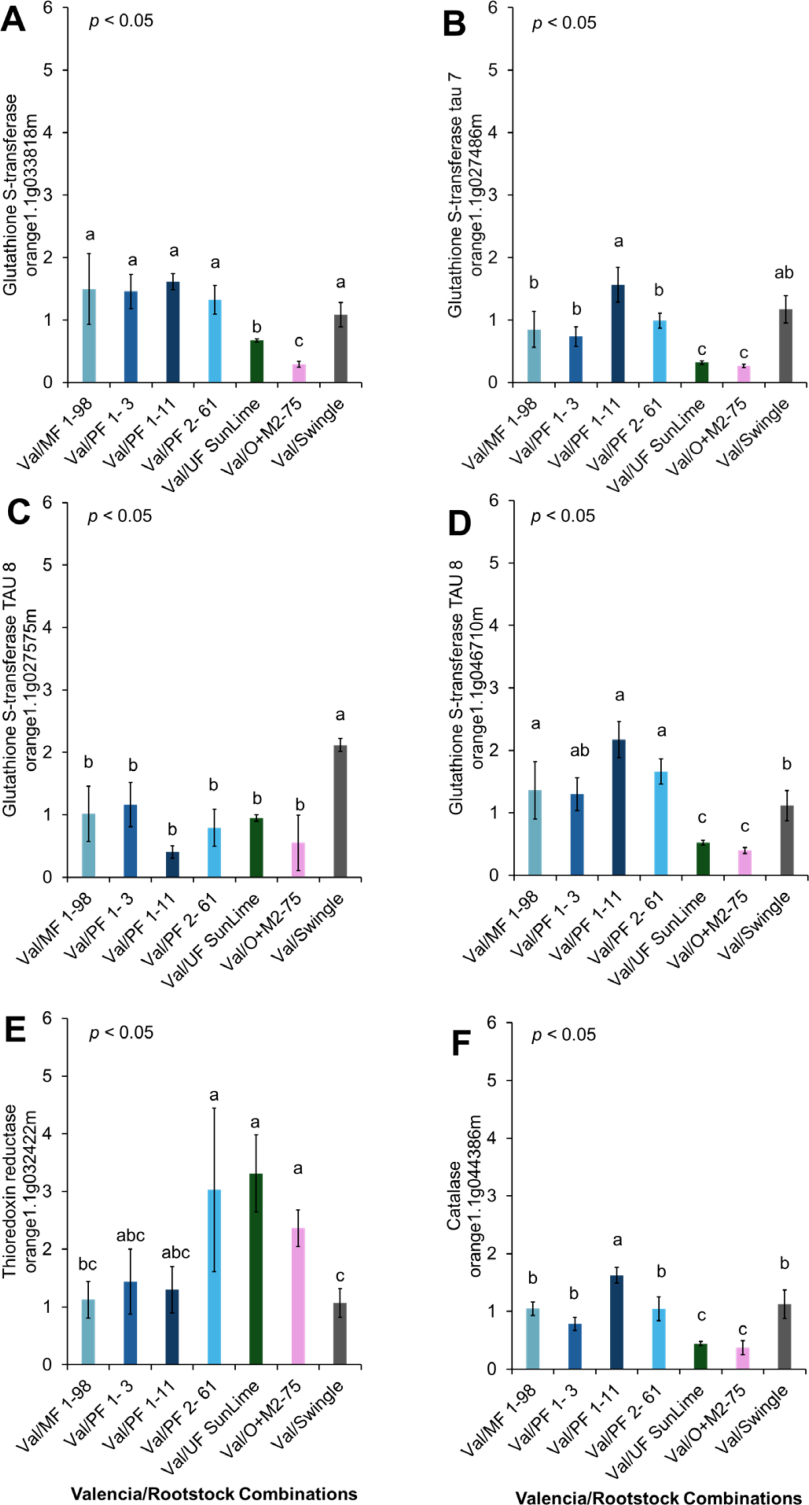
Relative expression of selected genes involved in detoxification and stress response. Different letters indicate significant differences across cultivars (p < 0.05). **(A)** Glutathione S-Transferases (orange1.1g033818m), **(B)** Glutathione S-Transferase Tau 7 (orange1.1g027486m), **(C)** Glutathione S-Transferase Tau 8 (orange1.1g027575m), **(D)** Glutathione S-Transferase Tau 8 (orange1.1g046710m), **(E)** Thioredoxin reductase (orange1.1g032422m), **(F)** Catalase (orange1.1g044386m).

**Figure 9:**
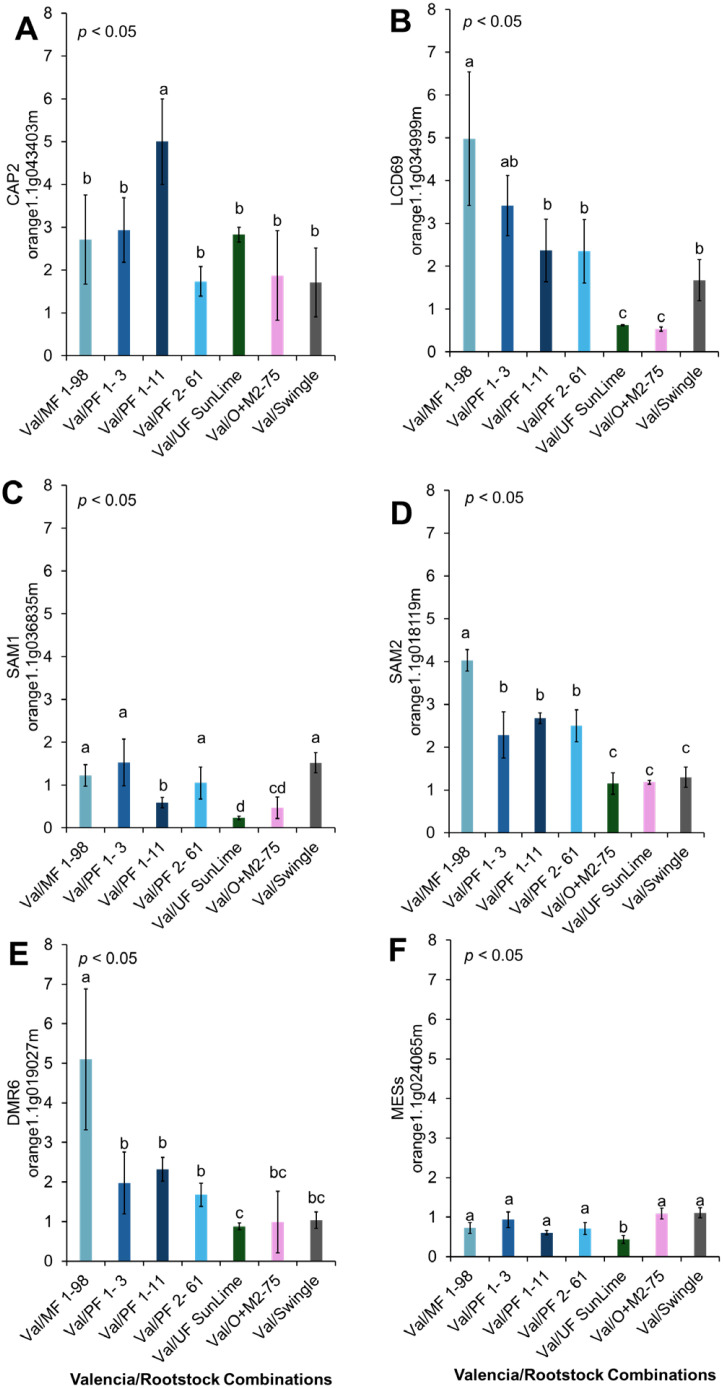
Relative expression of selected genes associated with defense. Different letters indicate significant differences across cultivars (p < 0.05). **(A)** PR1-like (CAP2: orange1.1g043403m), **(B)** LCD69 (orange1.1g034999m), **(C)** SAM1 (orange1.1g036835m), **(D)** SAM2 (orange1.1g018119m), **(E)** DMR6 (orange1.1g019027m), and **(F)** MES2 (orange1.1g024065m).

**Figure 10:**
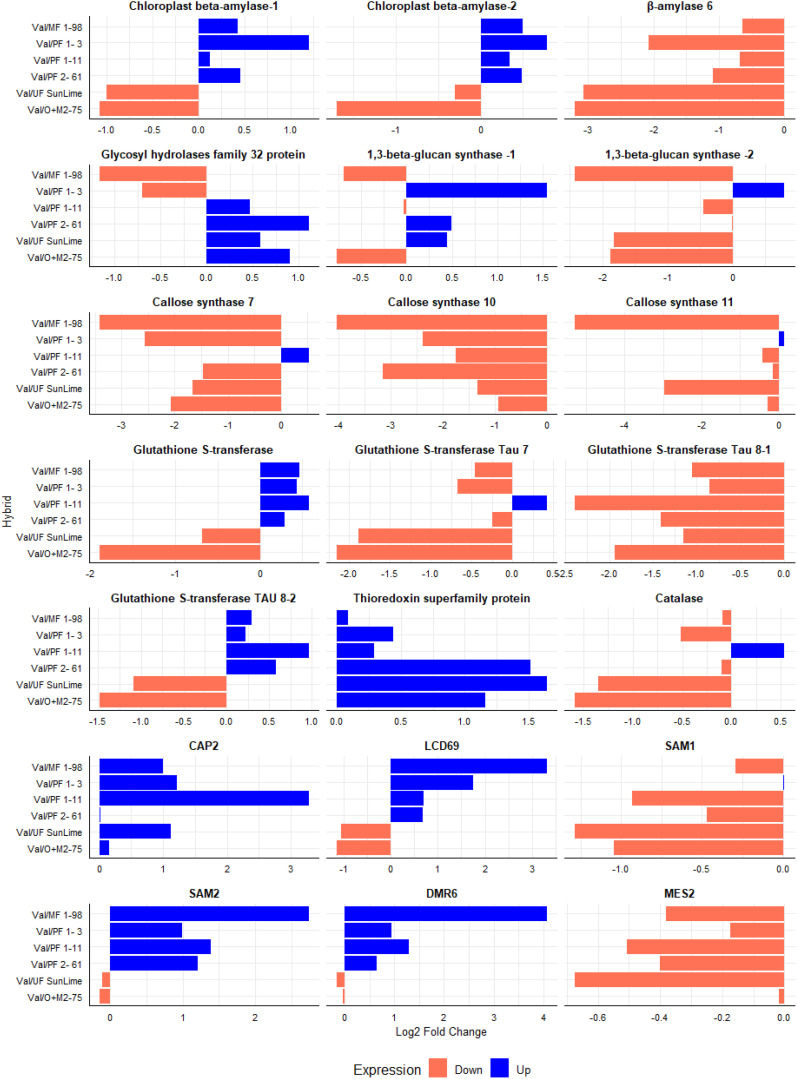
Comparison of Log2 fold change between ‘Valencia’ grafted onto Australian Lime derived hybrids compared to ‘Valencia’ grafted onto Swingle rootstock. The upregulation is presented in blue; the downregulation is presented in orange. The data was analyzed in response to *Ca*Las infection.

The relative expression levels of the selected genes were calculated using the 2^^−ΔΔCT^ method (**Figures 6**–**9**), with ‘Valencia’ grafted onto Swingle rootstock under *Ca*Las+ infection as the reference condition. To provide an integrated overview of gene expression responses, we additionally calculated and visualized the log2 fold change of selected genes involved in carbohydrate metabolism, cell wall synthesis, detoxification, and defense-related responses relative to the Swingle-grafted control (**Figure 10**).

The relative expression of genes involved in carbohydrate metabolism and cell wall synthesis in ‘Valencia’ grafted onto the hybrid rootstocks was compared with ‘Valencia’ grafted onto Swingle. These included genes encoding *Glycosyl hydrolase family 32 protein* (orange1.1g008242m), *Beta-Amylase* 6 (orange1.1g010067m), and *Chloroplast Beta-Amylase* (orange1.1g017328m). We observed upregulation of *Chloroplast Beta-Amylase* in Val/PFL1-3, Val/MFL1-98, Val/MFL 1–11 and Val/MFL 2–62 compared with Val/SW. Additionally, Val/PFL2–61 showed significant upregulation of *Glycosyl hydrolases* compared with Val/SW (**Figures 6**, **10**).

Val/PFL1–3 exhibited significant upregulation of the expression of *1,3-Beta-Glucan Synthase*, followed by Val/PFL1–11 and Val/PFL2-61 (**Figures 7A, B**, **10**). Furthermore, we analyzed the relative expression of *Callose Synthase 7* (orange1.1g037960m), *Callose Synthase 10* (orange1.1g000180m), and *Callose Synthase 11* (orange1.1g000258m). Val/PFL1–11 showed significant upregulation of *Callose Synthase 7* compared with Val/SW. ‘Valencia’ grafted onto Australian Lime-derived hybrids exhibited downregulation of the expression of *Callose Synthase 10.* However, Valencia’ grafted onto PFL1–3 exhibited higher *Callose Synthase 11* expression compared to Val/SW. In contrast, Val/MFL1–98 significantly reduced *Callose Synthase* gene expression compared with Val/SW (**Figures 7**, **10**).

The expression patterns of genes involved in detoxification and stress responses varied when ‘Valencia’ plants grafted onto different rootstocks were compared. *Glutathione S-Transferases (GSTs)*, including *Glutathione S-Transferase Tau 7*, and *Glutathione S-Transferase Tau 8*, showed slight differences in expression. *Glutathione S-Transferase and Glutathione S-Transferase Tau 8* (orange1.1g046710m) were highly expressed in Val/PFL 1-11, Val/PFL 2-61, Val/PFL 1-3, and Val/MFL1–98 compared with Val/SW. Moreover, the *Thioredoxin* protein superfamily (*Thioredoxin reductase*) exhibited significantly higher expression in ‘Valencia’ grafted onto Australian Lime Derived Hybrids compared to Val/SW. *Catalase* expression exhibited higher expression in Val/PFL 1–11 compared to Val/SW (**Figures 8**, **10**).

We investigated the relative expression of several defense-related genes, including *PR1-like* (*CAP2*)*, LCD69, SAM1, SAM2*, *DMR6*, and *MES2*, across different graft combinations. *CAP2* was highly upregulated in Val/PFL 1-11, Val/PFL 1-3, Val/MFL1-98, and Val/UF SunLime compared with Val/SW. The relative expression of *LCD69, SAM2, and DMR6* was highly expressed in Val/MFL1-98, Val/PFL 1-3, Val/PFL 1-11, Val/PFL 2-6; however, its levels were significantly lower in Val/O+M2–75 and Val/UF SunLime compared with Val/SW. *SAM1* and *CsMES2* showed relatively lower expression compared to Val/SW (**Figures 9**, **10**).

## 4 Discussion

Considerable efforts have been made to identify citrus varieties resistant to citrus greening disease due to its devastating economic impact and the challenges of disease management (Pérez-Hedo et al., 2024). The urgency of identifying reliable sources of resistance within *Citrus* and its relatives has intensified in recent years. There are numerous challenges associated with traditional breeding for the genetic improvement of citrus varieties to increase tolerance or resistance to *Ca*Las and HLB. These include long juvenile periods, nucellar embryony, sexual incompatibility, excessive heterozygosity from crosses, and male or female sterility in novel combinations (Talon and Gmitter, 2008). Despite these challenges, our breeding program has devoted significant efforts over the past decade to develop and assess hybrids derived from Australian limes. Many of these hybrids exhibit natural resistance or tolerance to *Ca*Las and serve as valuable genetic resources for increasing the resilience of commercial citrus varieties (Dutt et al., 2021; Dutt, 2022a, b; Mahmoud et al., 2024b, 2025). One promising strategy involves developing cultivars that can be utilized as rootstocks or scions, facilitating the integration of resistance traits into commercial production systems.

Using vigorous rootstocks in citrus cultivation is essential for growing healthy trees that produce high yields (Bowman and Joubert, 2020). These rootstocks can also enhance disease resistance, improve nutrient uptake, and increase tolerance to environmental stressors (Grosser et al., 2014; Mahmoud et al., 2021). Utilization of resistant rootstocks could increase citrus tree productivity and provide long-term economic benefits to growers despite the persistent threat of HLB (Castle et al., 2015). In our study, ‘Valencia’ sweet orange grafted onto Australian lime derived hybrids were graft compatible and exhibited variable effects on growth parameters, including scion growth, tree height, and trunk diameter. ‘Valencia’ plants grafted onto PFL2–61 and MFL1–98 presented increased vigor, increased height, and increased trunk diameter when compared to control trees. Trees infected with *Ca*Las demonstrated reduced scion growth compared with those grafted onto HLB-free rootstocks. The hybrids evaluated in this study exhibited compatibility with ‘Valencia’ sweet orange, except for PFL1-3. Despite its significant field resistance to HLB, PFL1–3 did not consistently form successful graft unions, demonstrating potential limitations in its use as a rootstock. Graft compatibility among Citrus and related genera is generally widespread within the Citrinae subfamily. However, not all genera exhibit successful grafting. Alves et al. (2021) reported that most genotypes grafted onto ‘Rangpur’ lime were compatible, including Citrus, Poncirus, Atalantia, *C. australasica*, *C. glauca*, and related hybrids. However, *Limonia acidissima* and *C. halimii* showed reduced graft compatibility. These incompatibility reactions were not associated with *Ca*Las infection, as uninfected controls presented similar symptoms, indicating that the grafting issues were inherent to the genotypes rather than induced by *Ca*Las infection.

We observed that, compared with those grafted onto the Swingle rootstock, ‘Valencia’ sweet orange trees grafted onto specific hybrid rootstocks, such as PFL1–11 and UF Sunlime, exhibited lower *Ca*Las titers after 24 months. However, ‘Valencia’ plants grafted onto PFL1-3, MFL1-98, or PFL2–61 presented higher *Ca*Las titers than those grafted onto Swingle. Also, the variation in pigment concentrations between *Ca*Las-infected and noninfected plants can be attributed to several physiological factors. The levels of chlorophyll *a* and *b*, essential components for photosynthesis, are often affected by plant health status. Infected plants typically experience physiological stress, which disrupts chlorophyll biosynthesis and maintenance. This disruption likely arises because the pathogen interferes with metabolic processes, leading to reduced chlorophyll production (Cheaib and Killiny, 2024). Conversely, the level of carotenoids, which play crucial roles in protecting plants from oxidative stress and photodamage, tends to increase in infected plants (Munné-Bosch, 2005). This shift in pigment composition may serve as an adaptive response to mitigate the detrimental effects of *Ca*Las-induced stress. Additionally, we observed significant variation in starch content following *Ca*Las infection. Leaves from ‘Valencia’ grafted onto PF 1–11 and Swingle presented the highest starch content. Furthermore, our findings indicate that the accumulation of phenolic and flavonoid compounds is primarily influenced by rootstock genotype and time, rather than *Ca*Las infection status alone. This is supported by our observation that certain non-infected genotypes, such as Val/Swingle, exhibited TFC and TPC levels comparable to or exceeding those in infected plants. Such overlaps may reflect constitutive biochemical profiles specific to individual rootstocks, which can mimic pathogen-induced changes. These results prove the complex interplay between host genotype, temporal dynamics, and pathogen response, with implications for understanding rootstock-mediated tolerance mechanisms. Following infection and *Ca*Las multiplication in phloem tissue, the plant’s defense system responds continually, leading to long-term physiological changes that further impair the plant’s health. One significant aspect of this prolonged response is the persistent overproduction of ROS, inducing oxidative stress throughout the plant (Ma et al., 2022; Weber et al., 2022). In addition to ROS accumulation, callose deposition occurs in phloem sieve tubes, contributing to systemic cell death in phloem tissue, including companion and sieve element cells (Weber et al., 2022; Welker et al., 2022). This progressive damage disrupts nutrients and signal transport (Folimonova et al., 2009; Kim et al., 2009; Mahmoud et al., 2023). Phloem disruption results in metabolic imbalance; in particular, it leads to starch accumulation in chloroplasts, which is associated with the downregulation of essential metabolic processes such as photosynthesis, respiration, and energy production (Suh et al., 2021). Consequently, the plant exhibits characteristic blotchy mottle symptoms on the leaves, a hallmark of HLB. This damage is especially evident in the branches, fruits, and roots of infected trees. While initially intended to control the infection, the chronic defense response ultimately leads to widespread tissue degeneration due to oxidative stress and disrupted hormonal signaling (Pérez-Hedo et al., 2024).

We observed significant upregulation of *Beta-Amylase 6* and *Chloroplast Beta-Amylase*, which play crucial roles in starch degradation, in MFL1-98. Similarly, increased expression of *1,3-Beta-Glucan Synthase* and *UDP-Glucose-1,3-Beta-D-Glucan Glucosyltransferase* was observed in PFL1–11 and PFL2-61. Additionally, callose synthase genes exhibited differential expression in ‘Valencia’ grafted onto the hybrid rootstocks. The breakdown of callose and starch through Glucan 1,3-beta-glucosidases could contribute to the availability of simpler sugars or intermediates. Callose deposition genes and Glucan 1,3-beta-glucosidases play crucial roles in plant defense mechanisms. Callose deposition genes are responsible for synthesizing callose, a β-1,3-glucan polysaccharide, which is deposited at the site of pathogen attack to slow pathogen invasion and spread (Li et al., 2023). On the other hand, Glucan 1,3-beta-glucosidases are enzymes that catalyze the hydrolysis of β-1,3-glucans, breaking down callose and other β-glucans (Cairns and Esen, 2010).

Secondary metabolites are critical for mitigating oxidative stress and strengthening structural barriers against pathogens. Phenolic compounds and flavonoids play crucial roles in plant defense via their antimicrobial and antioxidant properties, thereby mitigating the toxic effects of ROS (Tuladhar et al., 2021; Shen et al., 2022). Flavonoids are considered free-radical scavengers because of their high reactivity as hydrogen or electron donors (Cotelle, 2001; Pannala et al., 2001). They play dual roles by acting as antioxidants to neutralize ROS (Pietta, 2000; Agati et al., 2012; Shen et al., 2022) and by directly contributing to pathogen inhibition (Biharee et al., 2020; Wang et al., 2022). Compared with the other rootstocks, ‘Valencia’ grafted onto MFL1–98 presented the highest TPC and TFC. Additionally, PFL2–61 and MFL1–98 presented the highest expression of Glutathione S-Transferase Tau 8. Additionally, PFL2–61 presented the highest expression of Thioredoxin superfamily proteins. Thioredoxins are essential for maintaining the cellular redox balance and protecting against oxidative stress. Together, these two genotypes demonstrate complementary strengths, making both prime candidates for improving plant resilience.

Antimicrobial peptides are crucial for directly inhibiting pathogen growth to increase plant resistance under stress (Li et al., 2021a). These peptides include lipid transfer proteins, puroindolines, α-/β-thionins, γ-thionins, and plant defensins, all vital in defending plants against bacterial and fungal attacks. γ-Thionins, often called plant defensins because of their unique three-dimensional structures, play a key role in pathogen recognition and neutralization (Mignone, 2022; Roychoudhury et al., 2024). We recorded significant upregulation of low-molecular-weight cysteine-rich 69 (*LCD69;* orange1.1g034999m*)* and SAM-dependent carboxyl methyltransferase (*SAM2;* orange1.1g018119m) in MFL1-98, further revealing their role in disease resistance and stress responses. SAM-dependent carboxyl methyltransferase is an enzyme that catalyzes the transfer of a methyl group from S-adenosylmethionine (SAM) to a carboxyl group on various substrates, typically resulting in the formation of methyl esters. This methylation process plays a significant role in the regulation of plant growth, development, and defense mechanisms (Li et al., 2021b).

## 5 Conclusions

This study demonstrates the promising potential of Australian lime derived hybrids as rootstocks for managing HLB and improving citrus tree health. Hybrids such as PFL2–61 and MFL1–98 exhibited significant growth enhancement. Although graft compatibility issues were observed with certain hybrids, the overall resistance to *Ca*Las was significant, as evidenced by the reduced *Ca*Las titer and adaptive physiological responses. The variations in pigment concentrations and starch contents further confirmed the ability of the plants to cope with stress and maintain metabolic balance. These findings provide a basis for developing resilient citrus cultivars, offering a strategic approach for mitigating the impact of HLB on citrus farming.

## Data Availability

The original contributions presented in the study are included in the article/**Supplementary Material**. Further inquiries can be directed to the corresponding author.
