# Supplementary material for: Novel citrus hybrids incorporating Australian lime genetics: development of HLB-tolerant citrus rootstocks and physiological changes in ‘Valencia’ sweet orange scions
Source: Front Plant Sci. 2025 Jun 13;16:1614845. doi: 10.3389/fpls.2025.1614845 (PMC12202233; doi:10.3389/fpls.2025.1614845)
Supplement: Supplementary file 1 [file DataSheet1.pdf]

Supplementary Material

Novel Citrus Hybrids Incorporating Australian Lime Genetics: Development of HLB-Tolerant Citrus Rootstocks and Physiological Changes in ‘Valencia’ Sweet Orange Scions

Lamiaa M. Mahmoud[1], Manjul Dutt[1,2]*

[1] Citrus Research and Education Center, University of Florida, Lake Alfred, FL, USA

[2] Plant Breeding Graduate Program, University of Florida, Gainesville, Florida, USA

Supplementary Table 1. TAQMAN based primer sequences used to amplify a 87-bp fragment of the *Ca*Las rplJ/rplL ribosomal protein gene

Primer	Sequence (5' to 3')
CQUL-F	TGGAGGTGTAAAAGTTGCCAAA
CQUL-R	CCAACGAAAAGATCAGATATTCCTCTA
CQUL-Probe	ATCGTCTCGTCAAGATTGCTATCCGTGATACTAG

Supplementary Table 2. List of the primer sequences used in SYBR Green-based qPCR assay.

No.	Gene ID	Forward	Reverse
1	orange1.1g047891m	TAGCACGAATGCTGGCTAAA	CCGAACTAATCCTTCTGGTGAG
2	orange1.1g017328m	TTGACAGCCGGATACTACAAT AC	GGCTGTTCTCTGTCTCTCATTT
3	orange1.1g010067m	GCAGAGCTTACTGCAGGATAT TA	CCTCATCTCAAGACAGGTGAAA
4	orange1.1g008242m	CGTCAACCACTAACTTGTCCA	ACGACCGGCTGAATATTATCAC
5	orange1.1g045737m	GCAGCTTCAGCTAGCTACTATT	CCACGACCAGTTGCTCTATAC
6	orange1.1g001473m	CACACTCTCTATGTGGTTCCTG	CCAATCCGTCCAATCATCTACT
7	Orange1.1g037960m	AAGATGAGTCGAGCCCAAAC	CGACTCTTGGATTGTCCTTCTC
8	orange1.1g000180m	GGAAAGTTGCAGGAGGAAATG	CAGTGGTGAAGTAGAAGGAGAG
9	orange1.1g000258m	CCTAATGCCAGAAGAGCAGTT	GTATGCCAGACCAAGTCCATAC
10	orange1.1g033818m	CATGGCTTGGGAGAGAAGAA	AGCGAGGGAATTTGTGTGATA
11	orange1.1g027486m	GGAGCATGAGAAGGCGATAAA	TAGCAATCCATCCAAAGACGAG
12	orange1.1g027575m	CAGTTTGCGATTCCATGGTTATT	ACAGCAACTCCCTTGTCTTC
13	orange1.1g046710m	GGAGCAAAGGAGAGGAGAAAC	TCCAATTGCAAGGTCCAAATAAC
14	orange1.1g032422m	CGTCAACCACTAACTTGTCCA	ACGACCGGCTGAATATTATCAC
15	orange1.1g044386m	GGATGAGGAGGTCGATTACTTTC	CTTCTCACGCCTTCCTGTAAT
16	orange1.1g043403m	TGGGTGAATGAGAAAGCTGATTA	CGCCAAACCACCTGAGTATAG
17	orange1.1g034999m	CTACTTGTGCTGCTGCTTCT	AGCACAGTTGCTCTTACTCAC
18	orange1.1g036835m	TACCCGAGTTTCAAGTGTTTCT	GAACCTGGCATTCCTGCTAT
19	orange1.1g018119m	GAACTCACCTGCCTACAACA	CTTGTGCCCTGGCTAGTAAA
20	orange1.1g019027m	CCTCAG CCC GAT CTAACTTATG	CAGGAAGCCATTTCCCATTTC
21	orange1.1g024065m	AGCTGGTTGGACACTCAATTT	TCAGGAGGACAAAGCTGATAGA
